#  Age-Specific Gastric Cancer Risk Indicated by the Combination of *Helicobacter pylori* Sero-Status and Serum Pepsinogen Levels

**DOI:** 10.7508/ibj.2015.03.002

**Published:** 2015-07

**Authors:** Sana Eybpoosh, Yeganeh Talebkhan, Samaneh Saberi, Maryam Esmaeili, Akbar Oghalaie, Fatemeh Ebrahimzadeh, Toktam Karimi, Afshin Abdirad, Azin Nahvijou, Mohammad Ali Mohagheghi, Mahmoud Eshagh Hosseini, Marjan Mohammadi

**Affiliations:** 1 *HPGC Group, Dept. of Medical Biotechnology, Biotechnology Research Center, Pasteur Institute of Iran, Tehran, Iran;*; 2 *Research Center for Modeling in Health, Institute for Futures Studies in Health, Kerman University of Medical Sciences, Kerman, Iran;*; 3 *Cancer Institute, Tehran University of Medical Sciences, Tehran, Iran; *; 4 *Cancer Research Center, Tehran University of Medical Sciences, Tehran, Iran; *; 5 *Dept. of Gastroenterology, Amiralam Hospital, Tehran University of Medical Sciences, Tehran, Iran*

**Keywords:** Biomarkers, Demography, Age Distribution

## Abstract

**Background:**

Serologic screening of gastric cancer (GC) by serum pepsinogens (sPG) levels and Helicobacter pylori (Hp) sero-status, though highly informative, has provided heterogeneous results. Here, we have evaluated the modifying effects of demographic factors on the risk impact of Hp sero-status/sPG levels in gastric cancer, with particular emphasis on age.

**Methods:**

A cross-sectional study was carried out on 1341 individuals (GC = 578, healthy = 763), who were stratified into two age groups: 35-59 years (middle-aged, n = 830) and ≥ 60 years (60 years-plus, n = 511). Demographic factors and serological states (Hp sero-staus and sPG levels) were recorded by subject interview and serum ELISAs, respectively. Covariate-specific odds ratios were calculated by multivariable logistic regression.

**Results:**

Hp infection was consistently associated with increased sPGI and sPGII levels in the 60 year-plus, but not the middle-aged group. The joint examination of the variable states of the three serum biomarkers (Hp serology, sPGI, and sPGI/II ratio), in the 60 year-plus age group, demonstrated a stepwise escalation of risk from the single (sPGI_low_; OR = 2.6), to double (sPGI_low_/sPGI/II_low_; OR = 3.55, and Hp_positive_/sPGI_low_; OR = 5.0) and ultimately triple (Hp_positive_/PGI_low_/PGI/II_low_; OR = 10.48) positive states, in reference to the triple negatives. However, this pattern was not exhibited in the middle-aged subjects.

**Conclusion:**

Age was clearly identified as a modifying factor on the risk projection of the combined states of Hp serology and sPG levels in gastric cancer screening, reflected by the augmented (~10.5 fold) risk of GC in the triple positive (Hp_positive_/sPGI_low_/sPGI/II_low_) 60 year-plus subjects, which was not evident in the middle-aged group.

## INTRODUCTION

Based on the 2012 Press Release of the International Agency for Research on Cancer (IARC), gastric cancer (GC) is rated as the third leading cause of cancer mortality, resulting in an annual loss of more than 700,000 people to this mortal disease [1]. Chronic *Helicobacter pylori* (Hp) infection leading to atrophic gastritis is considered as a group I carcinogen, previously declared by this agency [2]. However, in reality, a vastly expanding list of additional host and environmental susceptibility factors come together to create grounds for a small fraction (1-3%) of Hp-infected subjects to develop GC [3]. GC is a silent killer that is often detected at terminal stages of the disease, after which the rate of five-year survival ranges from 4 to 27% from developing to developed countries, respectively [4]. Therefore, early screening, preferably by non-invasive and cost-effective methods, is a crucial need for risk surveillance. 

According to the Correa cascade [5], Hp infection triggers a cascade of histopathologic changes, which is initiated by chronic active gastritis, followed by atrophy, intestinal metaplasia, dysplasia, and eventually gastric adenocarcinoma (in a fraction of susceptible individuals). Development and progression of these histopathologic changes are monitored by means of endoscopy in some Asian countries [6, 7], but is not acceptable or applicable for most population screening programs. 

Gastric anatomy is classified into various sub-sections according to the composition of its secretory cells; hence, making it possible to track its histopathologic changes by tracing their secretory products, fluctuations of which are reflected in the serum. In this regard, the measurement of gastric pepsinogens, as the products of chief and antral pyloric glands, has long been introduced as a “serologic biopsy” method for tracing the histopathology of their producing cells, which would ideally mirror the histopathologic changes of the stomach in general and gastric atrophy and GC in particular [8]. In the face of their appealing features, most studies have reported less than acceptable and highly variable discrimination powers and risk indications for these biomarkers [9], which limit their utility as a screening tool. As Janes and Pepe [10] have noted, confounders may distort the diagnostic accuracy of biomarkers, if they associate with both the biomarker and outcome of interest. Despite this fact, few studies have focused on this issue and such data are particularly scarce from West Asian countries [2], where Hp infects the majority of the adult population [11].

Considering the fact that the initiative of population screening is taken by the screener not the target subjects, IARC’s handbook for cancer prevention [12], emphasizes on “strata-specific" application of screening strategies in order to maximize the benefits and minimize the emotional as well as financial expenses. In particular, this organization urges an age-specific method for cancer screening. 

As aging is an irrefutable risk factor for many cancers including GC [13], here, we have hypothesized that the vast heterogeneity in the discrimination powers of Hp/sPG (serum pepsinogens) method in different populations is partly due to varying demographic factors, particularly the age of the target population. Therefore, we have stratified our study population into two low- and high-risk age groups and used statistically sound methods to explore the power of sPGs and Hp sero-status as risk indicators of GC in each age stratum, while taking into account statistically and clinically significant demographic variables as confounding or effect modifying factors. 

## MATERIALS AND METHODS

***Subjects. ***We conducted a cross-sectional study with a comparison group. A convenience sampling method was used; all incident cases of histologically confirmed GC admitted to the National Cancer Institute (Tehran, Iran) were recruited during 2005-2013. Asymptomatic individuals who had referred for routine check-ups during the same time period and were ≥35 years of age were considered as the comparison group and will be hereafter referred to as “healthy” subjects. As the goal of this study was to explore the role of demographic variables in evaluating sPGs and Hp sero-status as risk indicators of GC, we did not match/restrict for these variables; instead, wherever a demographic variable was assumed to confound a relationship, its effect was controlled by the aid of regression modeling or stratification techniques [14, 15]. Written informed consents were obtained before data and sample collection according to the protocols approved by the National Committee on Ethical Issues in Medical Research, Ministry of Health and Medical Education of Iran; Ref No. 315.

***Interview data collection. ***Study participants were interviewed about demographic variables of interest using a structured questionnaire. Questions assessed participants’ age (in years), gender, ethnicity (Fars, non-Fars), smoking habit (never, ever [current or former], and passive), and family history of GC in the first degree relatives (yes, no). 

***Blood sample collection. ***Five milliliters of fasting venous blood were obtained from each subject, following provision of an informed consent and prior to the interview/surgery. Sera were isolated for measurement of anti-Hp IgG and sPGI and II levels. 

***Hp*** ***sero-status determination****.* Hp-specific IgG antibodies were detected by an in-house Hp IgG ELISA assay according to the previously described protocol [16]. Sera with titers above and below the defined cut-off points were considered as positive and negative, respectively. Samples with borderline titers were retested with the Hp IgG ELISA kit (Trinity Biotech, Ireland). Those remaining at borderline titers were not included in the statistical analyses.

***Serum pepsinogen measurements. ***sPG I and II levels were measured by ELISA kits (BIOHIT, Finland) according to the manufacturer’s instructions, and PGI/II ratio was calculated. Serum PG levels were considered as continuous format. For further assessments, we dichotomized serum PGI and PGI/II ratio based on commonly and commercially proposed cut-off values of 70 µg/l and 3.0, respectively [17]. We also studied various possible combinations of these three variables: Hp sero-status (positive/negative), sPGI (low/normal), and sPGI/II (low/normal) categories and included them in the regression models as independent risk indicators of GC. 

***Gastric tumor classification. ***To compare sPG levels between subcategories of gastric tumor and evaluate the role of demographic variables, we sub-classified cases of histologically confirmed gastric adenocarcinoma according to their: a) subsite (proximal/distal), b) histological subtype (intestinal/diffuse/mixed) based on Lauren’s classification [18], c) differentiation grade (well/moderate/poor) [19], and d) stage (early/late) according to TNM classification (T: primary tumor, N: regional lymph nodes, M: distant metastasis) [20]. 

***Statistical analysis. ***Continuous and categorical variables were described as mean (SD) and number (%), respectively. To evaluate the role of demographic variables on GC risk indication by sPGs, first we checked if serum PGI, PGII, and PGI/II ratio depend on demographic variables. For this purpose, we used multivariable linear regression (for sPGs) treating demographic variables as independent variables. While the effect of one demographic variable in these models was of interest, other demographic variables were considered as potential confounders and remained in the model, if their inclusion into the model changed the value of the coefficient of interest to more than 20% [21]. Demographic variables, whose distribution was significantly different between cases and healthy individuals, were forced into the regression models, regardless of the “20% rule” mentioned above. The distribution of serum PGs and their ratio was right-skewed and significantly deviated from normal distribution, based on Kolmogorov-Simonov test (*P* < 0.0001). Therefore, for linear regression modeling, where serum sPGs were considered as dependent variables, these variables were transformed into the normal distribution using logarithmic transformation. However, mean, SD, and adjusted regression coefficients (*ß*) are reported in numeric scale, using exponential back-transformation. The transformations provide regression equations, and thus regression coefficients (*ßs*) are in “multiplicative scale” and cannot be interpreted as conventional linear regression coefficients. In “multiplicative scale”, a regression coefficient shows the number of “times” the dependent variable changes per each unit change in the independent variable [22]. Our three GC risk indicators (PGI, PG I/II ratio, and Hp sero-status) were combined to generate a variable with all possible configurations: Then we used multivariable logistic regression models to estimate the odds of having GC, given the “combined variable” as the risk indicator of interest and age, gender, ethnicity, smoking and family history of GC as the stratifying variable. Serum PG levels were also compared between the strata of gastric tumor subcategories using multivariable linear regression as described previously. All analyses were done using Stata software (version 11). Results were considered as statistically significant at 0.05 levels. 

## RESULTS

**Fig. 1:**
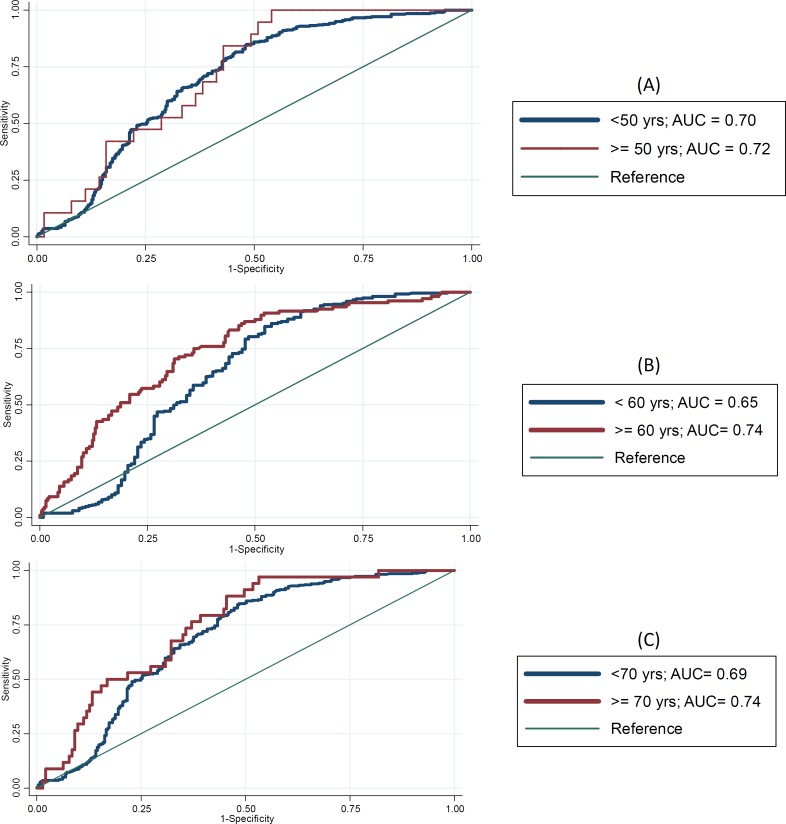
ROC curves of the discrimination power of serum PGI between different strata of age: upper and lower than 50 years (A); upper and lower than 60 years (B); upper and lower than 70 years (C).

***Age stratification. ***To determine the cut point for age stratification, we compared Receiving Operating Characteristic (ROC) curves of the discriminatory power (Area Under the Curve, AUC) of sPG levels between those older and younger than some commonly used cut points (50, 60, and 70 years old). Figure 1 shows ROC curves for each type of age stratification using serum PGI as the biomarker. It is evident that for PGI, the cut point at 60 year of age gives a better discrimination between GC and healthy individuals (*P* = 0.025; Fig. 1B). This result was also true for PGII (*P* = 0.028, data not shown). The AUC differences for the two age strata older and younger than 50 or 70 year cut points were not statistically significant (Fig. 1A and 1C). Therefore, our study population, which comprised of 1,341 individuals (GC = 578 [43.1%], healthy = 763 [56.9%]), were stratified into two age groups: 35-59 years (middle-aged, n = 830) and ≥ 60 years (60 years-plus, n = 511). Table 1 presents the distribution of demographic and tumor characteristics in GC and healthy subjects in each age stratum. 

***Association between serum PG levels and tumor characteristics. ***Since the discrimination power of serum PGs in detecting tumor subcategories did not differ between age cut points, their association was assessed without stratifying for age groups. This analysis showed that tumor subtype, staging, and grading did not significantly affect serum PG levels (Table 2). Stratification according to tumor location revealed that proximal tumors possessed lower sPGI and sPGII levels as compared with distal tumors, but did not reach statistical significance after adjustment for confounders (*P*_PGI_ = 0.074, *P*_PGII_ = 0.083). There was no difference observed in the measurement of sPGI/II ratio between different tumor subtypes (*P*_PGI/II _= 0.580). Therefore, for risk assessment, GC subjects were not sub-stratified based on tumor characteristics.

***Differences in serum pepsinogen levels between different demographic groups. ***Multivariable linear regression analysis, adjusting for all other variables, showed that varying patterns exist in sPG levels between categories of some demographic (age, gender, ethnicity, family history of GC, and smoking habits) and biologic (Hp sero-status) variables in GC patients and healthy individuals; some of which were found to be statistically significant (Table 3). 

In the middle aged group, mean sPGI/II ratio showed a significant decrease (20%) in males *vs.* females (*P*_Adjusted_ = 0.017), restricted to healthy subjects. On the other hand, the mean sPGI/II ratio in GC patients of this age group, with family history of GC, was 60% of those without family history (*P*_Adjusted_ = 0.027), which did not hold true for healthy subjects. Passive smoking reduced mean sPGII levels by 40% (*P*_Adjusted _= 0.031) again only in GC patients but not healthy subjects. This inconsistent pattern of association between demographic factors and sPG levels was not observed in the 60 year-plus age group.

Therefore, there was a coherent pattern of association between sPG levels (sPGI, sPGII, and sPGI/II ratio) and Hp infection in both cases and controls of the 60 year-plus group. However, a clear discrepancy was observed in the impact of Hp infection on sPG levels of cases as opposed to controls of the middle-aged group.

The highly critical variable affecting serum PG levels, in both age groups, was the Hp sero-status, which exhibited a very clear differential pattern. In the older age stratum, sPGI levels were significantly higher in Hp-positive GC (by 60%, *P*_Adjusted_ = 0.046) and healthy (by 50%, *P*_Adjusted_ = 0.022) subjects in reference to those with Hp-negative status. A significant elevation was also seen for the mean sPGII levels in Hp-positive GC subjects (by 30%, *P*_Adjusted_ = 0.027) as well as healthy ones (by 120%, *P*_Adjusted_ < 0.0001). Considering the levels of both sPGI and sPGII were increased as a result of Hp infection in the older age group, the sPGI/II levels remained unchanged in GC as well as healthy subjects.

**Table 1:** Distribution of demographic and tumor characteristics in the two age strata

**Variables**	**35-59 yrs**				**≥60 yr**s		
	**GC**		**Healthy**		**GC**		**Healthy**
	n (%)		n (%)		n (%)		n (%)
**Mean Age (SD)**	49.5 (8.1)		44.5 (9.5)		69.3 (6.2)		67.8 (7.3)
**Gender**							
* Female*	70 (31.8)		356 (58.4)		84 (23.5)		81 (52.9)
* Male*	150 (68.2)		254 (41.6)		274 (76.5)		72 (47.1)
**Ethnicity**							
* Fars*	53 (24.1)		270 (44.3)		79 (22.07)		68 (44.4)
* Non-Fars*	167 (75.9)		340 (55.7)		279 (77.93)		85 (55.6)
**Smoking status**							
* Never*	127 (57.7)		453 (74.2)		206 (57.6)		111 (72.6)
* Ever*	81 (36.8)		137 (22.5)		144 (40.2)		37 (24.2)
* Passive*	12 (5.5)		20 (3.3)		8 (2.2)		5 (3.2)
**Family history of GC**							
* No*	189 (85.9)		552 (90.5)		287 (80.2)		136 (88.9)
* Yes*	31 (14.1)		58 (9.5)		71 (19.8)		17 (11.1)
**Tumor Characteristics**							
**Subsite**							
* Proximal*	70 (31.8)		-		159 (44.4)		-
* Distal*	150 (68.2)		-		199 (55.6)		-
**Subtype**							
* Intestinal*	103 (46.8)		-		212 (59.2)		-
* Diffuse*	97 (44.1)		-		106 (29.6)		-
* Mixed*	20 (9.1)		-		40 (11.2)		-
**Stage**							
* Early*	24 (11.0)		-		38 (10.6)		-
* Late*	196 (89.0)		-		320 (89.4)		-
**Grade**							
* Poor*	121 (55.0)		-		153 (42.8)		-
* Moderate*	65 (29.6)		-		134 (37.4)		-
* Well*	34 (15.4)		-		71 (19.8)		-
**Total**	220 (100)		610 (100)		358 (100)		153 (100)

In the middle aged subjects, however, sPGI levels were only elevated in Hp-positive GC subjects (by 50%, *P*_Adjusted_ = 0.056) but not healthy patients. Conversely, the statistically significant rise in sPGII levels (by 80%, *P*_Adjusted_ < 0.0001) and drop in sPGI/II ratio (by 30%, *P*_Adjusted_ < 0.0001) were only observed in the Hp-positive healthy subjects, but not in GC patients in this age group.

***Age-specific combined effect of*** ***Hp*** ***sero-status with sPG levels on gastric cancer risk. ***Evaluation of the GC risk impact projected by the combined status of Hp/sPG by multivariable regression analysis, adjusting for age, gender, ethnicity, smoking, and family history of GC, produced data presented in Table 4. The joint examination of the variable states of the three serum biomarkers (Hp serology, sPGI, and sPGI/II ratio) created four categories: 1) triple negatives (reference group), 2) single positive, 3) double positives, and 4) triple positives.

In the 60 years-plus age group, a stepwise escalation of risk was sequentially observed for the single (sPGI_low_), double (sPGI_low_/sPGI/II_low_ and Hp_positive_/ sPGI_low_), and triple (Hp_positive_ sPGI_low_ sPGI/II_low_) positive subjects, yielding adjusted odds ratios ranging from 2.6 to 3.5-5.0 to 10.48, as compared with the triple negative reference subjects. A very different pattern, however, was observed in the middle aged group. In this age category, the joint assessment of the three biomarkers resulted in no statistically significant risk impact for either of the single, double, or triple positive groups of subjects, in reference to the triple negatives.

## DISCUSSION

Most cancers, particularly those of epithelial origin including that of the stomach, are age-related diseases. In light of prolonged exposure to a multitude of cancer-associated risk factors, aging allows for overtime accumulation of various molecular and physiological dysfunctions; namely genetic mutations, epigenetic changes, telomere dysfunction, etc. [23]. Acknowledging age-related distribution of cancers, IARC handbook of cancer prevention [12] recommends age-specific cancer screening programs to increase the efficiency of screening methods while avoiding undue emotional and financial expenses.

**Table 2:** Differences in mean serum PG levels between strata of tumor subcategories in GC patients

**Tumor Characteristics**	**PGI**			**PGII**			**PGI/II**	
	Mean (SD)	*P* value*		Mean (SD)	*P* value*		Mean (SD)	*P* value*
**Subsite**								
* Proximal *	31.7 (4.5)	Baseline		6.8 (2.8)	Baseline		5.0 (2.5)	Baseline
* Distal*	39.3 (3.7)	.074^a^		8.1 (2.9)	.083^b^		5.1 (2.6)	.580^a^
**Subtype**								
*Intestinal*	35.7 (4.7)	Baseline		7.8 (2.9)	Baseline		5.2 (2.2)	Baseline
*Diffuse*	40.1 (3.6)	.838^c^		7.2 (2.8)	.483^b^		5.1 (3.1)	.509^a^
*Mixed*	28.0 (3.3)	.269^c^		7.6 (3.1)	.477^b^		4.3 (2.6)	.549^a^
**Stage**								
* Late stage*	44.2 (3.4)	Baseline		7.9 (2.7)	Baseline		5.7 (2.5)	Baseline
*Early stage*	35.5 (4.1)	.565^c^		7.5 (2.9)	.906^c^		5.0 (2.6)	.175^a^
**Grade**								
*Poor*	36.1 (4.3)	Baseline		7.6 (3.1)	Baseline		5.3 (2.6)	Baseline
*Moderate *	35.5 (4.0)	.472^c^		7.5 (2.8)	.834^c^		4.6 (2.6)	.593^a^
*Well*	38.2 (3.9)	.474^c^		7.6 (2.8)	.224^c^		5.4 (2.5)	.662^a^

* *P* values were generated using multivariable linear regression. Potential confounders in multivariable regression coefficient were chosen based on statistical or clinical significance for each analysis.

a Adjusted for family history of GC (Yes/No), smoking (Never/ever/passive) and Hp serology (Positive/negative);

b Adjusted for^* a *^plus age (continuous format);

c Adjusted for ^*a*^ plus age, gender, and ethnicity.

GC incidence rises with age, holding a median diagnosis and mortality age of 69 and 72 years, respectively [24]. Common practice for serologic screening of GC [25] and that recommended by the commercial kits have long been the assessment of sPGI/II ratio. More recently, the recommendation of the ABC(D) method by the Japanese investigators [26, 27], has added the two variables of Hp sero-status and sPGI levels to the PGI/II ratio and categorizes subjects accordingly. Despite benefiting from a relatively high detection criteria, this method of categorization has faced highly variable projected risks even amongst the East Asian populations [28] and was not found applicable for some populations [29]. 

Aging creates a change of behavior which affects the performance criteria of potential biomarkers. We have, thus, hypothesized that the observed variability could be partly due to the age of the population. Accordingly, the difference between the power of discrimination (AUC) of sPGI and II was found significantly higher in the 60 year-plus *vs. *middle-aged subjects. In a study carried out in Taiwan, the rate of gastric intestinal metaplasia was found 2.66 higher in this age group as compared to younger subjects [30]. Similarly, the levels of candidate GC biomarkers such as miRNAs [31] were found significantly altered above this age cut-off. Furthermore, the informative value of certain genetic markers such as IL-1 beta [32] and H2 receptor [33] single nucleotide polymorphisms was drastically increased in subjects 60 years and older. In our study, serum PG levels were independently measured and compared between the different demographic strata in each age stratum. In particular, we focused on the impact of Hp sero-status on sPG levels amongst the two differing age groups, while taking into account the effect modifying factors of age, gender, ethnicity, family history of GC, and smoking status. 

Multivariable logistic regression analysis revealed a clear segregation of risk behaviors between the two age groups. Accordingly, Hp infection resulted in a consistent rise in sPGI and sPGII levels with no effect on PG I/II ratio, in both GC and healthy subjects of the 60 year-plus category, whereas a variable pattern was observed in the middle-aged category. The consistent effect of Hp infection on sPG levels in cases and controls of the older age category produced a logical stepwise escalation of the projected risk from the single to double and ultimately triple positive state(s) in reference to the triple negatives. Our observed stepwise increase in odds ratio follows the Korean [34] and not the Japanese [35] pattern, in the sense that Hp_postitive_PG_low_ (OR = 10.48) subjects were at greater risk of GC than Hp_negative_PG_low_ (OR = 3.55) individuals.

The above mentioned behavior was not however observed in the middle-aged group, which we speculate was due to inconsistent effect of Hp infection on sPG levels amongst cases and controls of this age group.

**Table 3:** Multivariable linear regression of demographic and biologic variables on mean serum PGs

**Independent Variables**	**PGI**						**PGII**						**PGI/II ratio**				
	**GC patients**			**Healthy individuals**			**GC patients**			**Healthy individuals**			**GC patients**			**Healthy individuals**	
	*ß*	*P *value		*ß*	*P *value		*ß*	*P *value		*ß*	*P *value		*ß*	*P *value		*ß*	*P *value
**35-59 yr**s																	
**Age **(in years)	1.0	.077		1.0	.652		1.0	.125		1.0	.049		1.0	.142		1.0	<0.0001
**Gender **																	
* Male vs. female*	1.1	.823		1.1	.264		0.9	.735		1.2	.019		1.4	.129		**0.8**	**.017**
**Ethnicity **																	
* Fars vs. non-Fars*	1.1	.698		1.0	.721		1.1	.514		1.1	.161		0.8	.296		1.0	.969
**Family history of GC**																	
*Yes vs. no*	0.7	.226		1.0	.748		1.2	.378		0.8	.095		**0.6**	**.040**		0.9	.885
**Smoking status **																	
* Ever vs. never*	0.9	.705		1.1	.200		1.0	.896		1.1	.560		0.8	.237		1.0	.625
* Passive vs. never*	0.5	.099		1.2	.460		0.8	.423		**0.6**	**.031**		0.7	.320		1.1	.521
Hp** serology **																	
*Positive vs. negative *	1.5	.056		1.1	.414		1.3	.093		**1.8**	**<0.0001**		0.9	.630		**0.7**	**<0.0001**
																	
**≥60 yrs**																	
**Age **	1.0	.476		1.0	.038		1.0	.738		1.0	.834		1.1	.165		1.0	.176
**Gender **																	
* Male vs. female*	1.2	.485		0.9	.904		1.2	.279		1.3	.135		0.9	.565		1.1	.514
**Ethnicity **																	
* Fars vs. non-Fars*	1.2	.457		1.2	.200		1.1	.495		1.2	.217		1.3	.156		1.0	.936
**Family history**																	
* Yes vs. no*	1.1	.591		1.0	.975		1.1	.342		0.9	.609		0.9	.254		1.1	.711
**Smoking status **																	
* Ever vs. never*	1.1	.571		1.1	.775		1.0	.889		0.7	.092		1.0	.860		1.1	.711
* Passive vs. never*	0.7	.554		0.8	.596		0.9	.700		0.6	.216		1.1	.827		1.2	.768
**Hp** ** s** **erology **																	
* Positive vs. negative *	**1.5**	**.046**		**1.5**	**.022**		**1.3**	**.027**		**2.2**	**<0.0001**		1.0	.732		0.9	.447

Regression coefficients (*ß*) and their corresponding *P* values were generated using multivariable linear regression, treating serum PGs as dependent variables and demographic and biologic variables as independent variables; each independent variable was adjusted for the rest of the independent variables presented in this Table. Statistically significant values are bolded.

**Table 4:** Multivariable logistic regression of Hp sero-status, PG I, and PG I/II on GC Odds: by categories of age

**Risk variables**	**GC**	**Healthy**		**OR (95% CI)**			
	n (%)	n (%)		Crude	*P* value	Adjusted a	*P* value
**35-59 yr**s							
Hp serology, sPG I and sPG I/II*							
***Triple Negative *** Hp _negative_ PGI _normal _PGI/I _normal_	26 (11.8)	88 (14.4)		1	-	1	-
***Single Positive*** Hp _negative_PGI _low_ PGI/II _normal_	30 (13.6)	69 (11.3)		1.5 (.8, 2.7)	.216	1.6 (.8, 3.1)	.179
***Single Positive*** Hp _negative_PGI _normal_ PGI/II _low_^b^	0 (0.0)	0 (0.0)		-	-	-	-
***Double Positive*** Hp _negative_ PGI _low_ PGI/II_low_	9 (4.1)	6 (1.0)		**5.1 (1.7, 15.6)**	**.005**	2.2 (.7, 7.2)	.190
*** Single Positive*** Hp _positive_ PGI _normal _PGI/II _normal_	58 (26.4)	287 (47.1)		.7 (.4, 1.2)	.153	0.6 (.3, 1.0)	.061
***Double Positive*** Hp _positive_ PGI _low_ PGI/II _normal_	74 (33.6)	132 (21.6)		**1.9 (1.1, 3.2)**	**.016**	1.8 (1.1, 3.2)	.062
***Double Positive*** Hp _positive_ PGI _normal_ PGI/II _low_^b^	3 ( 1.4)	1 (0.2)		-	-	-	-
***Triple Positive*** Hp _positive_ PGI _low_ PGI/II _low_	20 (9.1)	27 (4.4)		**2.5 (1.2, 5.2)**	**.013**	1.4 (.7, 3.1)	.379
**Total**	220 (100)	610 (100)					
							
**≥60 yrs**							
Hp serology, sPG I and sPG I/II*							
***Triple Negative *** Hp _negative_ PGI _normal _PGI/II _normal_	26 (7.3)	22 (14.4)		1	-	1	-
***Single Positive*** Hp _negative_PGI _low_ PGI/II _normal_	40 (10.9)	17 (11.1)		1.9 (.9, 4.3)	.106	**2.6 (1.1, 6.3)**	**.034**
***Single Positive*** Hp _negative_PGI _normal_ PGI/II _low_^b^	1 (0.3)	1 (0.7)		-	-	-	-
***Double Positive*** Hp _negative_ PGI _low_ PGI/II _low_	18 (5.0)	5 (3.3)		3.1 (1.0, 9.5)	.056	**3.55 (1.1, 11.9)**	**.040**
***Single Positive*** Hp _positive_ PGI _normal _PGI/II _normal_	87 (24.3)	70 (45.8)		1.1 (.6, 2.0)	.879	1.6 (.8, 3.2)	.199
***Double Positive*** Hp _positive_* PGI *_low_* PGI/II *_normal_	131 (36.0)	29 (19.0)		**3.8 (1.9, 7.6)**	**<.0001**	**5.0 (2.3, 10.8)**	**<.0001**
***Double Positive*** Hp _positive_ PGI _normal_ PGI/II _low_^b^	2 (1.4)	3 (1.8)		-	-	-	-
***Triple Positive*** Hp _positive_ PGI _low_ PGI/II _low_	53 (14.8)	6 (3.9)		**7.5 (2.7, 20.7)**	**<.0001**	**10.48 (3.5, 31.1)**	**<.0001**
**Total**	358 (100)	153 (100)					

* The cut-offs used for dichotomization of PGI and PGI/II ratio were 70 µg/l and 3.0, respectivcely.

a adjusted for age (continuous), gender, ethnicity (Fars/non-Fars), smoking (Ever/never, passive) and family history of GC (Yes/no).

b Due to small cell sizes, crude and adjusted CIs (Confidence Interval) and ORs (Odds Ratio) are not calculated for these categories. Statistically significant values are bolded.

Therefore, the lower than expected detection rates reported by a pooled meta-anlysis for the use of serum PG in GC screening [25] may be partly owed to the lack of age group classification.

 We, in accordance to others [34], observed that Hp infection and aging have a contrasting effect of rising *vs.* lowering sPG levels, respectively. On the other hand, both of these covariates are repeatedly proven to be confirmed risk factors for GC [36, 37]. While aging cannot be reversed, the modifying role of Hp infection on sPG levels is demonstrated by decreased levels of sPG I and II following Hp eradication [34]. 

The observed discrepancy between the two age groups may also be due to the reported likelihood that Hp-induced inflammation [38], hyper-secretion of glands [39], and subsequent elevation of their secretory products, including sPGs (particularly sPGII) compensate for their reduction from the atrophic foci in the younger subjects. Therefore, the conflicting impact of these two covariates (aging and Hp infection) on sPG levels, if not taken into account, may counteract and mask the actual role of sPG levels as GC biomarkers. Therefore, stratification for age and Hp infection is recommended prior to and in addition to statistical adjustment for these covariates as confounders [40].

The observation of such drastic differences of behaviors between the two age groups, while supports the application of all three variables in the risk screening of older (60 years-plus) subjects, it cautions their informative value in GC screening of the middle-aged subjects, particularly for low-income countries.

Previous reports on the odds ratio of GC in various Hp-sPG categories have been quite heterogeneous [Reviewed in 8]. This matter could be due to the fact that demographic factors such as age, gender, ethnicity, family history of GC, and smoking habits have rarely been taken into account in the statistical models. Hence, their roles as confounders or effect modifiers may have masked or over represented the actual risk impact, which could also explain the varying and suboptimal diagnostic accuracies observed in different studies [41]. Our study, in agreement with the pertinent reports, reviewed by Kim and Jung [8], clearly demonstrated that these factors should be carefully monitored and incorporated into the statistical analysis, as they not only affect sPG levels, but also independently amplify the risk of GC. Having controlled for these covariates, we have been able to unmask the actual risk impact, which was found substantially higher than previously reported odds ratio [8, 42, 43] and exceeded 10 fold in the triple positive older aged subjects. 

Having performed tumor sub-classification, however, we did not find any significant differences in sPG levels between different strata of tumor subtypes, grades, or stages. Only sub-stratification according to tumor location, demonstrated a marginally lower levels of sPG levels in proximal *vs.* distal tumors, which could be due to the impaired function of chief cells caused by these tumors in the gastric corpus [17].

The strengths of our study include the analysis of a fairly large sample population and rigorous statistical analysis, controlling for most possible confounding and effect-modifying covariates. Selection of the end stage of the disease (GC) rather than the predisposing histopathologic changes (including atrophy), as the selected outcome provides: 1) the advantage of having a clear confirmed diagnosis (of GC) *vs.* the doubtful detection of patchy atrophic foci; and 2) the disadvantage of questionable applicability of the findings to the early screening capacity of these serum biomarkers. 

In summary, considering the world population is aging, which gives rise to previously undetected incidence of cancers, it is crucial to separately investigate senior (60 years-plus) individuals and devise age-specific recommendations, which may not necessarily apply to younger subjects. In our study, having carefully controlled for potential confounders, the triple positive (Hp_positive_/sPGI_low_/sPGI/II_low_) state was found highly informative for subjects over the age of 60, who seemed at a significantly greater risk of GC. Further evaluation of this hypothesis in longitudinal prospective studies will help ascertain its validity.
